# Population pharmacokinetics of penicillin G: insights into increased clearance at low concentrations to guide development of improved long-acting formulations for syphilis and prevention of rheumatic fever

**DOI:** 10.1128/aac.00269-25

**Published:** 2025-05-20

**Authors:** Okhee Yoo, Sam Salman, Thel K. Hla, Joshua Osowicki, Madhu Page-Sharp, Julie A. Marsh, Renae Barr, Kristy Azzopardi, Michael Morici, Kevin T. Batty, Stephanie L. Enkel, Joseph Kado, Lara Hatchuel, Alma Fulurija, James S. McCarthy, Thomas Snelling, Andrew C. Steer, Jonathan Carapetis, Laurens Manning

**Affiliations:** 1 Wesfarmers Centre for Vaccines and Infectious Diseases, The Kids Research Institute 117610, Nedlands, Western Australia, Australia; 2 Pharmacy, School of Allied Health, University of Western Australia 569809, Perth, Western Australia, Australia; 3 Institute for Paediatric Perioperative Excellence, University of Western Australia 2720, Perth, Western Australia, Australia; 4 Centre for Optimisation of Medicines, School of Allied Health, University of Western Australia, Perth, Western Australia, Australia; 5 Medical School, University of Western Australia, Crawley, Western Australia, Australia; 6 Clinical Pharmacology and Toxicology Unit, PathWest, Perth, Western Australia, Australia; 7 Department of Infectious Diseases, Fiona Stanley Hospital 418838, Murdoch, Western Australia, Australia; 8 Tropical Diseases Research Group, Murdoch Children’s Research Institute, Melbourne, Victoria, Australia; 9 Department of Paediatrics, University of Melbourne 2281, Melbourne, Victoria, Australia; 10 Infectious Diseases Unit, Department of General Medicine, The Royal Children’s Hospital Melbourne, Melbourne, Victoria, Australia; 11 Curtin Medical School, Curtin University 614117, Bentley, Western Australia, Australia; 12 Curtin Health Innovation Research Institute, Curtin University, Bentley, Western Australia, Australia; 13 Linear Clinical Research, Perth, Western Australia, Australia; 14 The Doherty Institute, University of Melbourne, Melbourne, Victoria, Australia; 15 Sydney School of Public Health, University of Sydney 117637, Sydney, New South Wales, Australia; 16 Perth Children's Hospital 60081, Nedlands, Western Australia, Australia; Providence Portland Medical Center, Providence Portland Medical Center, Portland, Oregon, USA

**Keywords:** benzylpenicillin, penicillin G, benzathine penicillin G, pharmacokinetics, cystatin C, eGFR, rheumatic heart disease, rheumatic fever, syphilis, renal clearance, population PK modeling

## Abstract

Although benzylpenicillin (penicillin G) is listed by the World Health Organization as an Essential Medicine, dose optimization is a persistent challenge, especially for long-acting intramuscular formulations. Maintaining sustained antibiotic exposure at target concentrations is crucial for secondary chemoprophylaxis of rheumatic heart disease and treatment of syphilis. This study compared the pharmacokinetic profile of continuous low-dose benzylpenicillin infusions with a standard-dose bolus and evaluated which renal function marker (serum creatinine, cystatin C, or combined e-glomerular filtration rate [eGFR]) best predicted clearance. Healthy adult volunteers received a single 600 mg IV benzylpenicillin bolus followed by randomization to continuous infusions targeting steady-state concentrations of 3, 6, 9, 12, or 20 ng/mL. Plasma benzylpenicillin concentrations were measured by liquid chromatography–mass spectrometry. Population pharmacokinetic analysis was performed using NONMEM by incorporating both bolus and infusion data, and various GFR estimations were evaluated as covariates for clearance. Data from 72 participants were analyzed, including 504 bolus and 389 continuous infusion samples. A two-compartment model improved fit when the ratio of central volume of distribution between bolus and low-dose infusion was incorporated, and clearance differences at steady state plasma concentration of 3 ng/mL were accounted for. Of the GFR estimations, cystatin C-based eGFR significantly enhanced model fit compared with creatinine-based equations. Benzylpenicillin pharmacokinetics at very low concentrations demonstrated both a higher volume of distribution and increased clearance. Cystatin C–based eGFR may more accurately predict benzylpenicillin clearance, enabling precision dosing for long-acting preparations used for treatment of syphilis and prevention of rheumatic fever.

## INTRODUCTION

Benzylpenicillin (penicillin G) is a narrow-spectrum beta-lactam antibiotic widely used against various gram-positive pathogens, including *Streptococcus* and *Staphylococcus* species and *Treponema pallidum*. As an intravenous preparation, it remains the cornerstone of therapy for pneumonia, meningitis, and endocarditis (1). Because of its low oral bioavailability, benzylpenicillin is typically administered intravenously as a sodium salt. By contrast, benzathine benzylpenicillin (benzathine penicillin G; BPG) is a long-acting intramuscular (IM) formulation where sustained low penicillin concentrations enable reduced dosing frequency. After IM injection, BPG is slowly hydrolyzed to benzylpenicillin, which is measurable in the plasma. BPG is used to prevent complications of *Streptococcus pyogenes* infections, such as acute rheumatic fever and rheumatic heart disease (RHD) (1, 2). For the estimated 30 million people worldwide affected by rheumatic fever and RHD (3), IM BPG administered every 3–4 weeks remains the only effective intervention for prevention of recurrence (1). It is also the standard treatment for syphilis, caused by *T. pallidum* with an estimated 7.1 million new infections reported in 2020 (4). In practice, for both RHD and syphilis, pain associated with IM BPG injections significantly hampers adherence, impacting clinical effectiveness. Additionally, global access and supply face substantial challenges, as most people around the world do not have reliable access to high-quality formulations. These challenges have prompted interest in developing a formulation that reduces injection discomfort and frequency, sustains therapeutic concentrations for both RHD and syphilis, and remains cost-effective. Informing the target product profile for a new formulation, data from a human challenge trial have recently demonstrated that concentrations above 9 ng/mL will prevent nearly all episodes of *S. pyogenes* pharyngitis. During this trial, we observed that at very low target steady-state plasma concentrations (Css), concentrations fell below predicted levels (5). This observation accords with data from recent studies of high-dose subcutaneous (SC) administration of BPG where an apparent rapid decline in plasma concentrations toward the end of the dosage interval suggests a differential in benzylpenicillin clearance at low concentrations (6).

Historically, pharmacokinetic (PK) studies of benzylpenicillin have focused on conventional high-dose intermittent or continuous infusion strategies (7, 8), and although more recent investigations have targeted specific populations (9–11), significant knowledge gaps persist, particularly concerning whether benzylpenicillin clearance changes at very low plasma concentrations (5).

Renal excretion is the most important pathway for benzylpenicillin clearance, involving both glomerular filtration and active tubular secretion. Dosing adjustments for renal impairment have traditionally been informed by creatinine clearance. For instance, prescribing information (BenPen) recommends that Clearance (mL/min) = 35.5 + 3.35 × Creatinine Clearance (mL/min), with a maintenance dose (g/24 h) = Clearance (mL/min) × Desired Serum Penicillin Concentration (µg/mL) × 0.00138 (12). However, emerging data indicate that cystatin C-based equations outperform creatinine-based methods such as Cockcroft–Gault in predicting renal drug clearance (13, 14).

In light of these considerations, in the present study, we address two primary objectives: (i) to investigate whether the pharmacokinetic profile of continuous low-dose benzylpenicillin infusions differs from a standard-dose bolus dose, particularly at very low target concentrations, and (ii) to evaluate whether serum creatinine (SCr), cystatin C (CysC), or combined estimated glomerular filtration rate (eGFR) measures more accurately predict benzylpenicillin clearance when accounting for additional potential covariates. This knowledge will inform the characteristics of new formulations of BPG and may ultimately guide more precise and individualized benzylpenicillin dosing strategies for RHD and syphilis where sustained, low plasma concentrations drive clinical efficacy.

## RESULTS

### Participants

The demographics of the study participants are summarized in Table 1. For the bolus dose group, a total of 72 participants were included, contributing 504 blood samples (seven samples per participant). For the continuous infusion group, 47 participants were randomized into target Css values as follows: nine participants for 3 ng/mL, 9 for 6 ng/mL, 8 for 9 ng/mL, 8 for 12 ng/mL, and 9 for 20 ng/mL.

**TABLE 1:** Demographic and baseline characteristics of study participants (*n* = 72)^*a*^

	Mean (%RSD)	Median (IQR [range])
Female number (%)	30 (42)	30 (42)
Age, years	27 (19)	25 (22–29 [18–38])
Weight, kg	71 (18)	69 (62–76 [50–107])
Height, cm	170 (6)	170 (164–177 [144–192])
Body mass index, kg/m^2^	24 (14.8)	24 (21–27 [18–32])
Body surface area, m^2^	1.80 (10.5)	1.79 (1.69–1.92 [1.49–2.35])
Serum creatinine, µmol/L	70 (19.3)	70 (61–78 [44–101])
Cystatin C, mg/L	0.60 (16.8)	0.59 (0.55–0.66 [0.39–0.87])

^*a*^ RSD denotes relative standard deviation.

Demographic and baseline characteristics of the 47 participants enrolled in the CHIPS trial and received continuous infusions of benzylpenicillin, stratified by assigned target Css (3, 6, 9, 12, and 20 ng/mL), are summarized in Table 2. A total of 389 blood samples were collected for PK analysis. The mean infusion rates increased proportionally with the target concentrations, as expected. However, considerable inter-individual variability in infusion rates was required within each group, reflecting differences in individual drug clearance. The groups were generally well-balanced in terms of age, weight, height, and BMI. Mean and median serum cystatin C concentrations were similar across all groups.

**TABLE 2:** Baseline characteristics of healthy volunteers enrolled in the CHIPS trial according to assigned target benzylpenicillin concentrations^*a*^

Assigned target concentration (ng/mL)		3	6	9	12	20	Total
Included in analysis		10	9	10	9	9	47
Number of plasma samples		80	66	86	77	80	389
Infusion rate, µg/h	Mean (%RSD)	87.1 (34.6)	218.3 (28.3)	340.6 (18.1)	364.1 (49.3)	640.3 (37.9)	332.1 (65.6)
	Median (IQR [range])	80.3 (60.4–108.2 [54.0–136.7])	220.4 (180.4–232.7 [131.4–331.0])	347.1 (287.9–384.7 [250.0–418.2])	367.5 (339.4–393.8 [286.6–448.1])	592.4 (514.3–620.6 [382.4–1076.5])	313.3 (180.9–411.2 [46.9–1133.6])
Female number (%)		6 (60)	4 (44)	5 (50)	6 (67)	6 (67)	27 (57)
Age, years	Mean (%RSD)	30 (17)	26 (19)	25 (23)	25 (21)	27 (17.3)	27 (20)
	Median (IQR [range])	29 (28–32 [20-38])	25 (21–29 (20–34])	23 (22–29 [18-35])	24 (23–27 [18-36])	28 (23–29 [19–33])	27 (22–30 [18-38])
Weight, kg	Mean (%RSD)	68 (19)	74 (12)	76 (20)	68 (23)	74 (17)	72 (18)
	Median (IQR [range])	67 (61–70 [50-99])	73 (71–78 [57-91])	70 (64–87 [60-107])	66 (58–70 [52-104])	71 (67–75 [60-97])	69 (63–75 [50-107])
Height, cm	Mean (%RSD)	172 (6)	171 (8)	172 (7)	172 (5)	169 (5)	171 (6)
	Median (IQR [range])	167 (164–178 [161-189])	169 (162–184 [151-188])	175 (169–178 [144-186])	172 (167–177 [157-181])	170 (162–176 [157-178])	170 (164–177.5 [144-189])
Body mass index, kg/m^2^	Mean (%RSD)	23 (17)	25 (11)	26 (17)	23 (16)	26 (13)	25 (15)
	Median (IQR [range])	22 (20–27 [18–29])	25 (25–28 [21–30])	25 (22–29 [20–32])	22 (21–23 [20–32])	26 (24–27 [21–31])	25 (21–27 [18–32])
Body surface area, m^2^	Mean (%RSD)	1.79 (10)	1.86 (10)	1.88 (12)	1.79 (12)	1.85 (9)	1.83 (11)
	Median (IQR [range])	1.74 (1.71–1.83 [1.53–2.23])	1.86 (1.78–1.95 [1.52–2.16])	1.85 (1.73–2.04 [1.57–2.31])	1.78 (1.66–1.85 [1.52–2.24])	1.84 (1.74–1.91 [1.60–2.13])	1.82 (1.72–1.94 [1.52–2.31])
Serum creatinine, µmol/L	Mean (%RSD)	62 (16)	74 (21)	70 (15)	70 (24)	67 (14)	69 (19)
	Median (IQR [range])	63 (56–69 [46-78])	76 (67–81 [44-101])	71 (65–77 [54-86])	70 (54–78 [46-94])	70 (61–70 [49-81])	70 (61–78 [44-101])
Cystatin C, mg/L	Mean (%RSD)	0.61 (16)	0.61 (20)	0.60 (17)	0.58 (15)	0.59 (10)	0.60 (17)
	Median (IQR [range])	0.61 (0.52–0.66 [0.50–0.81])	0.64 (0.61–0.66 [0.39–0.82])	0.59 (0.56–0.60 [0.48–0.86])	0.58 (0.56–0.59 [0.42–0.75])	0.59 (0.56–0.62 [0.49–0.69])	0.59 (0.55–0.66 [0.39–0.87])

^*a*^ RSD denotes relative standard deviation.

### Exploratory data analysis

**Fig 1:**
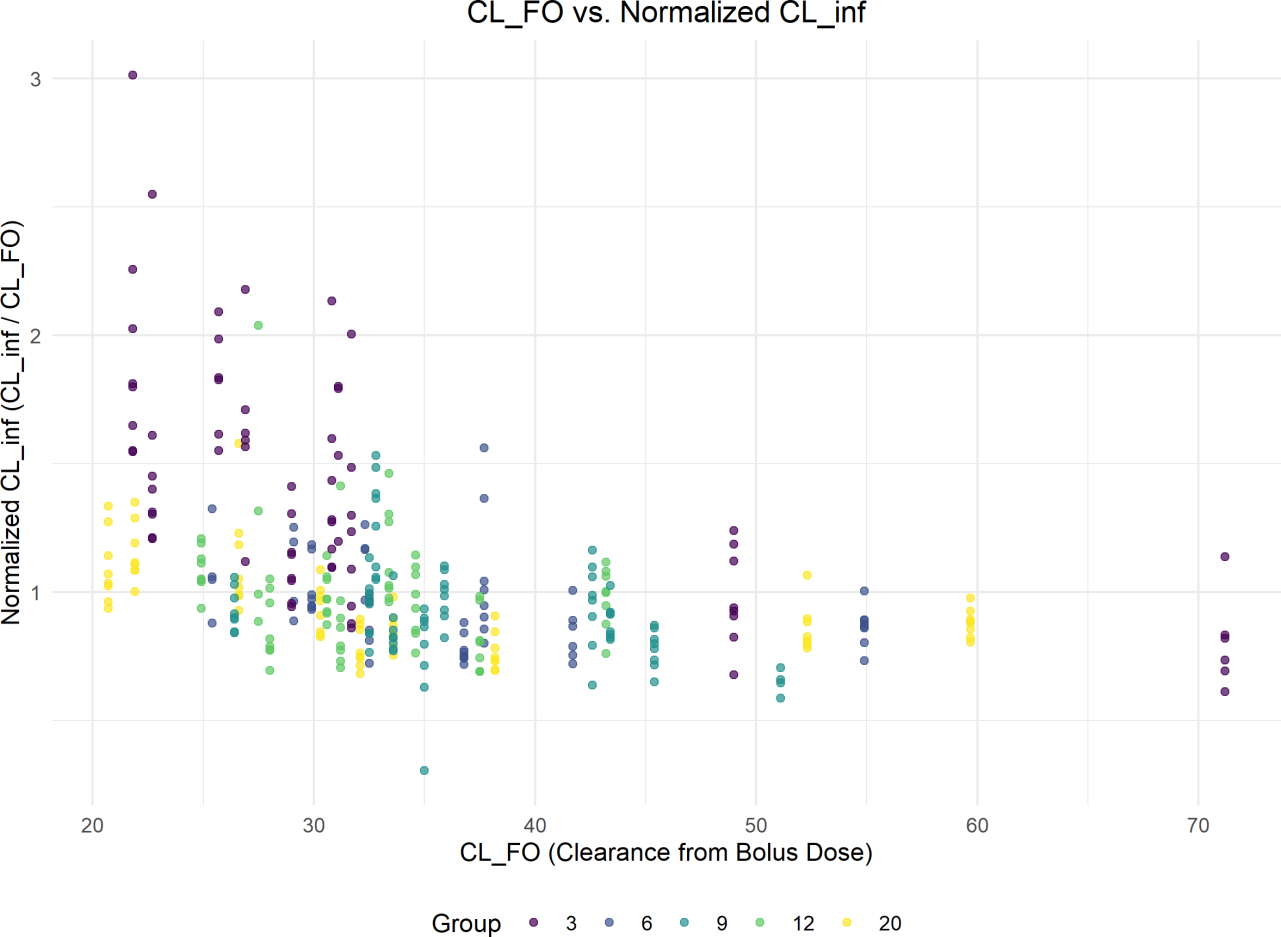
Discrepancies between benzylpenicillin clearance (L/h) estimates from bolus dose (CL_FO) and continuous infusion (CL_inf), particularly at lower CL_FO. The normalized steady-state clearance (CL_inf/CL_FO) is calculated by dividing the continuous-infusion clearance by the bolus-based clearance. Points are colored by target steady-state concentration group (3, 6, 9, 12, and 20 ng/mL).

The relationship between benzylpenicillin clearance estimated from the initial bolus dose (CL_FO) and the normalized steady-state clearance (CL_inf/CL_FO) during continuous infusion is shown in Fig. 1. Each Cl-FO corresponds to multiple steady-state clearance (CL_inf) values, as each infusion bag was administered, and plasma concentrations were measured every 12 h. A single Cl-FO had multiple corresponding normalized CL-inf/CL-FO values, which were represented as vertical dots in Fig. 1. These values were tightly clustered, except for those at a Css of 3 ng/mL, which exhibited a wider distribution. The Pearson correlation coefficient of –0.37 indicates a weak negative correlation between the two measures. The higher CL_inf compared with baseline clearance (CL_FO) indicates a nonlinear clearance pattern, which is more pronounced at a Css of 3 ng/mL. Of note, the plot demonstrates high variability, particularly in the group targeted to a Css of 3 ng/mL, suggesting greater fluctuations in clearance estimates at lower infusion rates.

**Fig 2:**
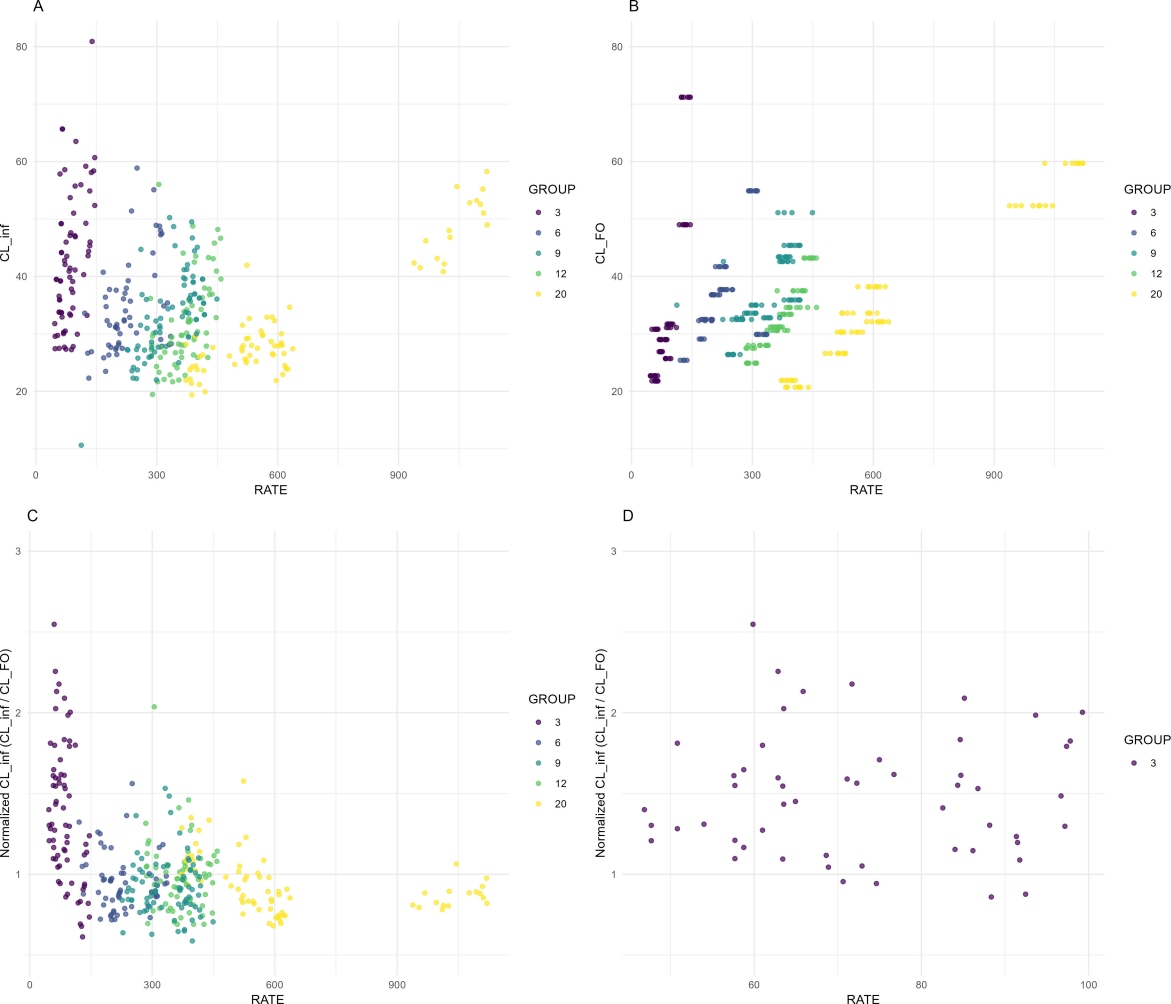
Relationship between infusion rate (RATE, µg/h) and benzylpenicillin clearance (L/h). (**A**) Steady-state clearance (CL_inf) vs. RATE. (**B**) Clearance estimated from a single intravenous bolus 600 mg dose (CL_FO) vs. RATE. (**C**) Normalized steady-state clearance (CL_inf/CL_FO) for rates below 1200 vs. RATE. (**D**) Normalized steady-state clearance (CL_inf/CL_FO) for rates below 100 m vs. RATE. Points are colored by target steady-state concentration group (3, 6, 9, 12, and 20 ng/mL). Data are from the CHIPS trial, a double-blind, placebo-controlled, randomized trial of benzylpenicillin for the prevention of experimental pharyngitis.

The CL_inf showed a similar trend to CL_FO as expected, as illustrated in Fig. 2A and B. Hence, when CL-inf is normalized to CL-FO, the values are expected to cluster around 1, indicating comparable clearance estimates. Although most data points are centered around a normalized clearance of 1, there is a tendency for normalized clearance to show higher variability, with CL-inf often exceeding CL-FO, particularly at lower infusion rates (Fig. 2C). A closer examination of the data, focusing on the group targeted to a Css of 3 ng/mL, highlights substantial variability in normalized clearance at low infusion rates, with several points exceeding 2 or approaching 3, suggesting that steady-state clearance was significantly higher than clearance estimated from the initial bolus dose in some individuals (Fig. 2D).

### Population pharmacokinetic analysis

Bolus and infusion data were fitted simultaneously, and a two-compartment model provided the best description of the data. Incorporating the ratio of central volume of distribution (Vc) between bolus and low-dose continuous infusion improved model fit (ΔOFV = −93.077). Further refinement, accounting for differences in CL at a target Css of 3 ng/mL, improved the fit further (ΔOFV = −157.818). Applying an allometric scaling factor of 0.75 for weight to account for differences in CL at a target Css of 3 ng/mL did not improve the model fit, as evidenced by a 2.589 increase in objective function value (OFV).

Including the ratio of clearance between bolus and low-dose continuous infusion significantly enhanced the fit (ΔOFV = −110.623). However, the model tended to overpredict concentrations at times less than 5 h and at the 97.5th percentile of visual predictive check (VPC) predictions. The difference in clearance was modeled by stratifying the infusion rate at 100 µg/h; however, the estimated difference in clearance was negative, indicating lower clearance at lower infusion rates, which contradicted the findings from the exploratory data analysis (EDA). Additionally, the stratified VPC demonstrated overprediction at a target Css of 3 ng/mL. Introducing zero-order elimination improved predictions at the 97.5th percentile but did not adequately address overprediction at times shorter than 5 h; hence, it was excluded.

Inter-individual variability (IIV) terms for clearance, central, peripheral volumes of distribution, and CL differences were included, with an additive error model best explaining residual variability. The relative standard errors (RSE%) for model parameters were all below 20%, and IIV estimates were below 17% except for the difference in clearance at lower target Css, which was nearly 40%. Weight was included as a covariate *a priori*, but additional covariates such as body surface area (BSA), age, and sex showed no significant relationship.

Glomerular filtration rate (GFR) estimates were evaluated as markers of total clearance of benzylpenicillin to determine which equation best aligned with benzylpenicillin clearance. The base model (without covariates) had an OFV of −1877.913 (Table 3). Among the tested GFR estimation equations, only eGFRcys significantly improved the model fit compared with the base model (OFV = −1889.986, ΔOFV = −12.073, *P* < 0.05). In contrast, incorporating CLcr or eGFRcr individually worsened the model fit (ΔOFV = +30.274 and +14.727, respectively), and the eGFRcr-cys model failed to provide further improvement. Overall, creatinine-based estimates did not adequately explain benzylpenicillin clearance.

**TABLE 3:** Comparison of estimated glomerular filtration rate models for benzylpenicillin clearance

Model	OFV	⊿OFV
Base model	−1877.913	
CLcr	−1847.639	+30.274
eGFRcys	−1889.986	−12.073
eGFRcr	−1863.186	+14.727
eGFRcr-cys	not converge	

The table shows the OFV for the base model and for models with different clearance equations added. ΔOFV indicates the change in OFV compared with the base model. A negative ΔOFV indicates an improvement in fit. The model with eGFRcr-cys did not converge. CLcr, creatinine clearance; eGFRcys, estimated glomerular filtration rate based on cystatin C; eGFRcr, estimated glomerular filtration rate based on creatinine; eGFRcr-cys, estimated glomerular filtration rate based on creatinine and Cystatin C.

The final model parameters, presented in Table 4, demonstrate good precision and robustness, with narrow bootstrap confidence intervals and low RSE% values for key structural parameters (CL, Vc, Q, and Vp). The bootstrap median OFV was slightly lower than the initial OFV, further indicating that the model is relatively stable and reliable.

**TABLE 4:** Final population pharmacokinetic model parameters and bootstrap results for benzylpenicillin^*a*^

Parameter	Mean	RSE%	Bootstrap median [95% CI]
CL, l.h^−1^ = θ × eGFRcys/60			
θ	13.3	2	13.31 [12.77–13.92]
V_C_, l.70kg^−1^	14.6	3	14.63 [13.79–15.54]
Q, l.h-1.70kg-1	5.34	5	5.33 [4.76–5.92]
V_p_, l.70kg^−1^	5.77	4	5.77 [5.33–6.25]
Ratio,_Vc_ where V_c,Linf_ = Vc × Ratio,_Vc_	1.96	4	1.96 [1.83–2.11]
Difference,_CL,Css3_, l.h^−1^ where CL_Css3_ = CL + Difference,_CL,Css3_	12.1	19	12.24 [7.85–16.92]
Interindividual Variability [shrinkage%]			
CL	14.4 [6]	10	14.3 [11.3–17.5]
V_C_	16.9 [22]	12	16.7 [12.3–20.5]
V_p_	13.7 [15]	11	13.6 [10.2–16.4]
Difference,_CL,Css3_	42.9 [69]	28	40.0 [13.5–63.5]
Residual Errors			
Proportional error (%)	16.5 [10]	1	16.5 [15.3–17.7]

^*a*^ CI, confidence interval; CL, population value of total clearance for an individual with eGFRcys = 60 ml/min; V_C_, population central volume of distribution for an individual with a body weight of 70 kg; Q, population value of intercompartmental clearance for an individual with a body weight of 70 kg; V_p_, population peripheral volume of distribution for an individual with a body weight of 70 kg; Ratio,_Vc_, ratio of the central volume of distribution between bolus infusion and low continuous infusion; V_c,Linf_, population central volume of distribution at low dose infusion for an individual with a body weight of 70 kg; Difference,_CL,Css3_, difference in the CL at a steady state target concentration = 3 ng/ml^−1^; CL_Css3_, population value of total clearance at a steady state target concentration of 3 ng/ml^−1^ for an individual with eGFRcys = 60 ml/min; % RSE, relative standard error [%RSE = 100 * (standard error/parameter estimate)]; inter-individual variability and residual error are presented as 100%$\times \sqrt{\text{variability}}\;\text{estimate}$.

**Fig 3:**
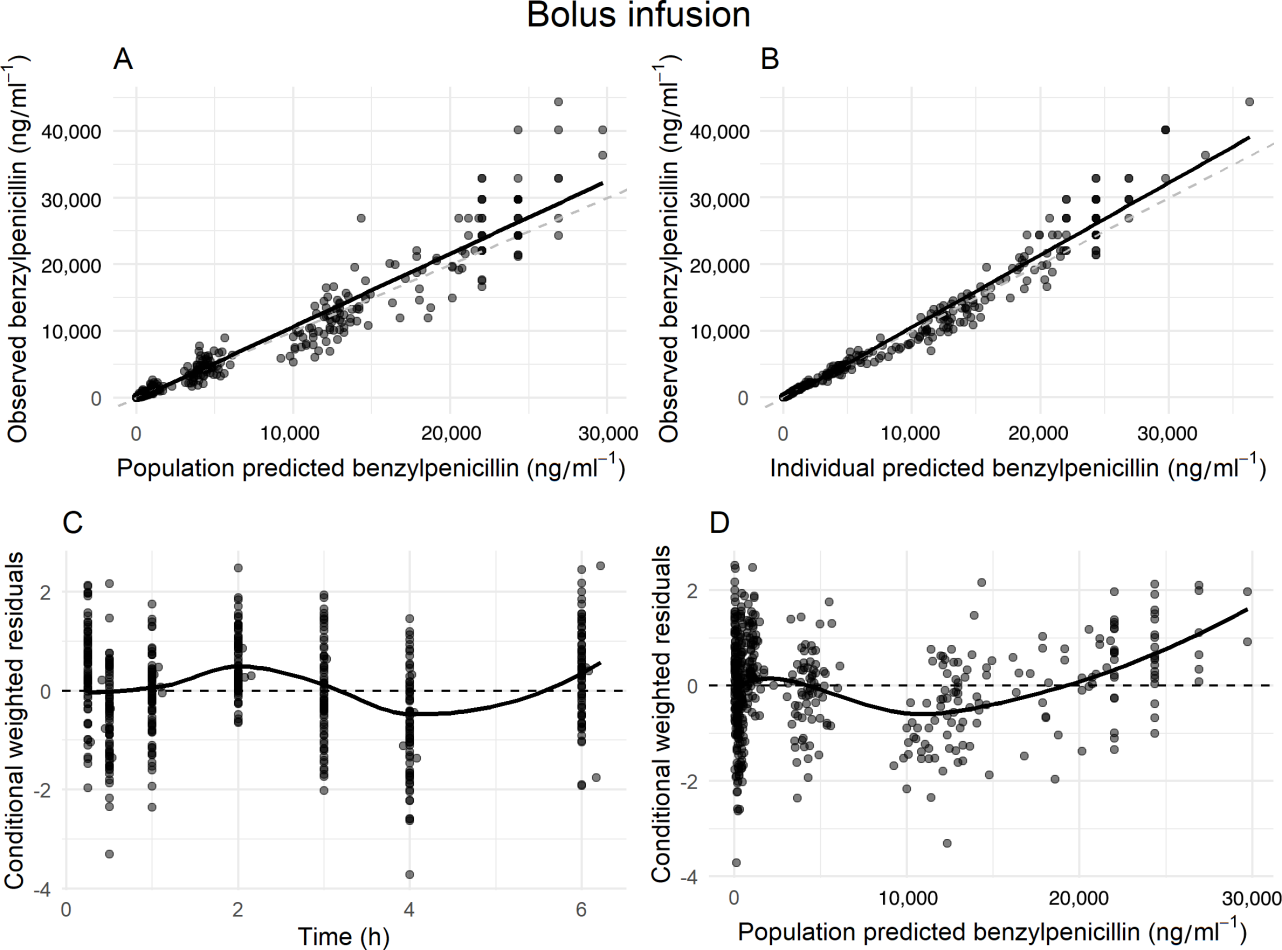
Diagnostic plots for the population pharmacokinetic model of bolus infusion. (**A**) Observed versus population-predicted benzylpenicillin concentrations, (**B**) Observed versus individual-predicted benzylpenicillin concentrations, (**C**) conditional weighted residuals versus time, and (**D**) conditional weighted residuals versus population-predicted concentrations. The dashed lines represent the lines of identity, whereas the solid lines indicate the lines of best fit.

**Fig 4:**
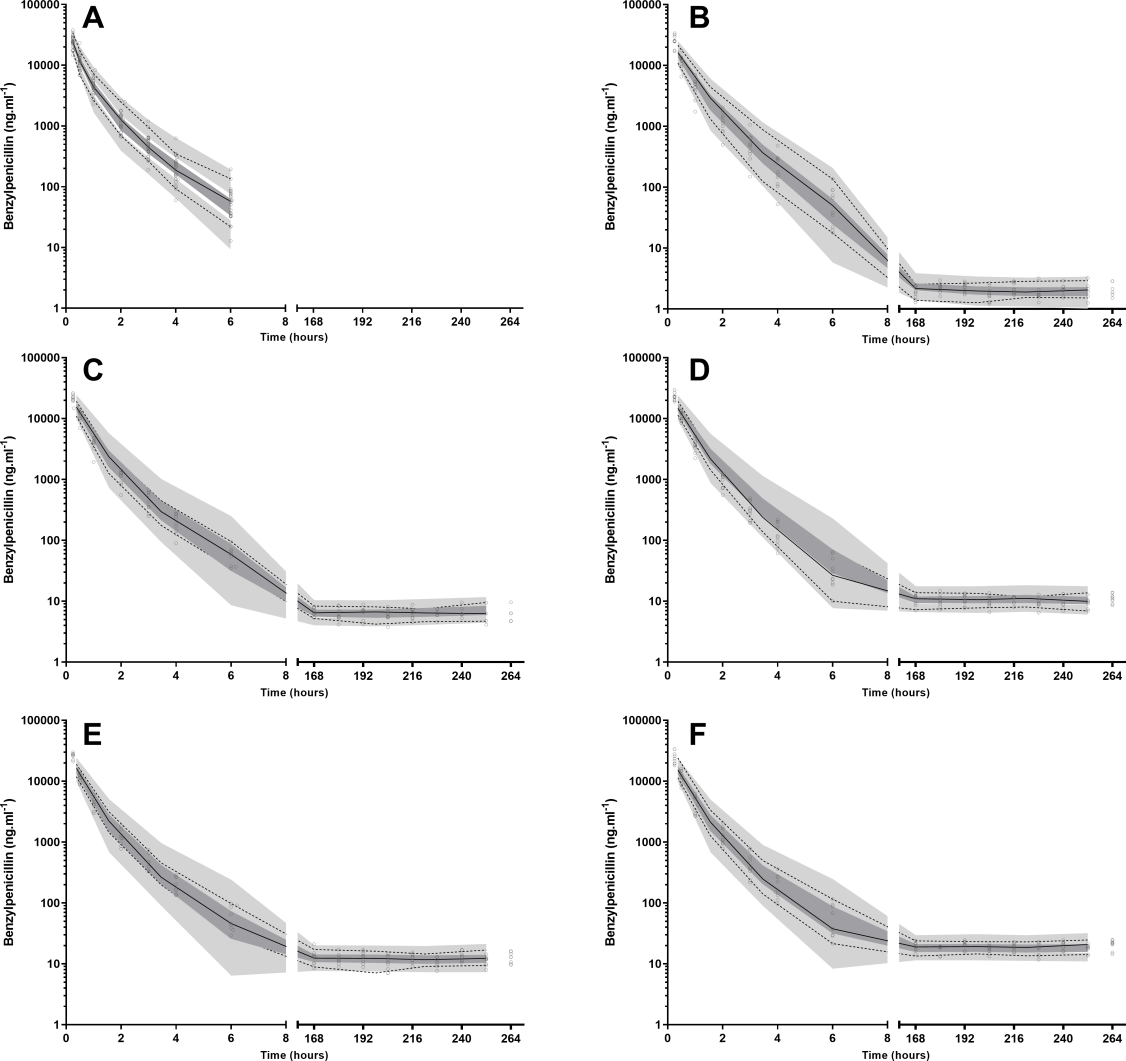
Visual predictive checks for benzylpenicillin plasma concentrations following a 600 mg bolus infusion and subsequent low-dose continuous infusions. (A) displays data from bolus doses only. (B–F) include both bolus and low-dose infusion data and correspond to target steady-state concentrations of 3, 6, 9, 12, and 20  ng/mL, respectively. Open circles represent the measured plasma concentrations of benzylpenicillin. Solid lines denote the median values of the observed data, whereas dashed lines indicate the 5th and 95th percentiles of the observed data. Shaded areas illustrate the 95% confidence intervals for the simulated values generated by the pharmacokinetic model, with the upper and lower shaded areas corresponding to the 95th and 5th percentiles, respectively, and the middle shaded area representing the 50th percentile.

The goodness-of-fit (GOF) plots of bolus infusion suggest that the model reasonably captures the central tendency of the data, with observed versus predicted plots (Fig. 3A and B) demonstrating good overall agreement and minimal outliers. However, conditional weighted residuals versus time (Fig. 3C) and population predictions (Fig. 3D) reveal positive residuals at higher concentrations, potentially reflecting limitations in capturing the distribution phase due to sparse data, with only a single observation at 15 min. This may inadequately characterize the early distribution phase, which is likely completed in 30 min. Similar trends are observed in the VPC, where the model slightly underpredicts early time points but shows a good fit for the remaining data (Fig. 4).

**Fig 5:**
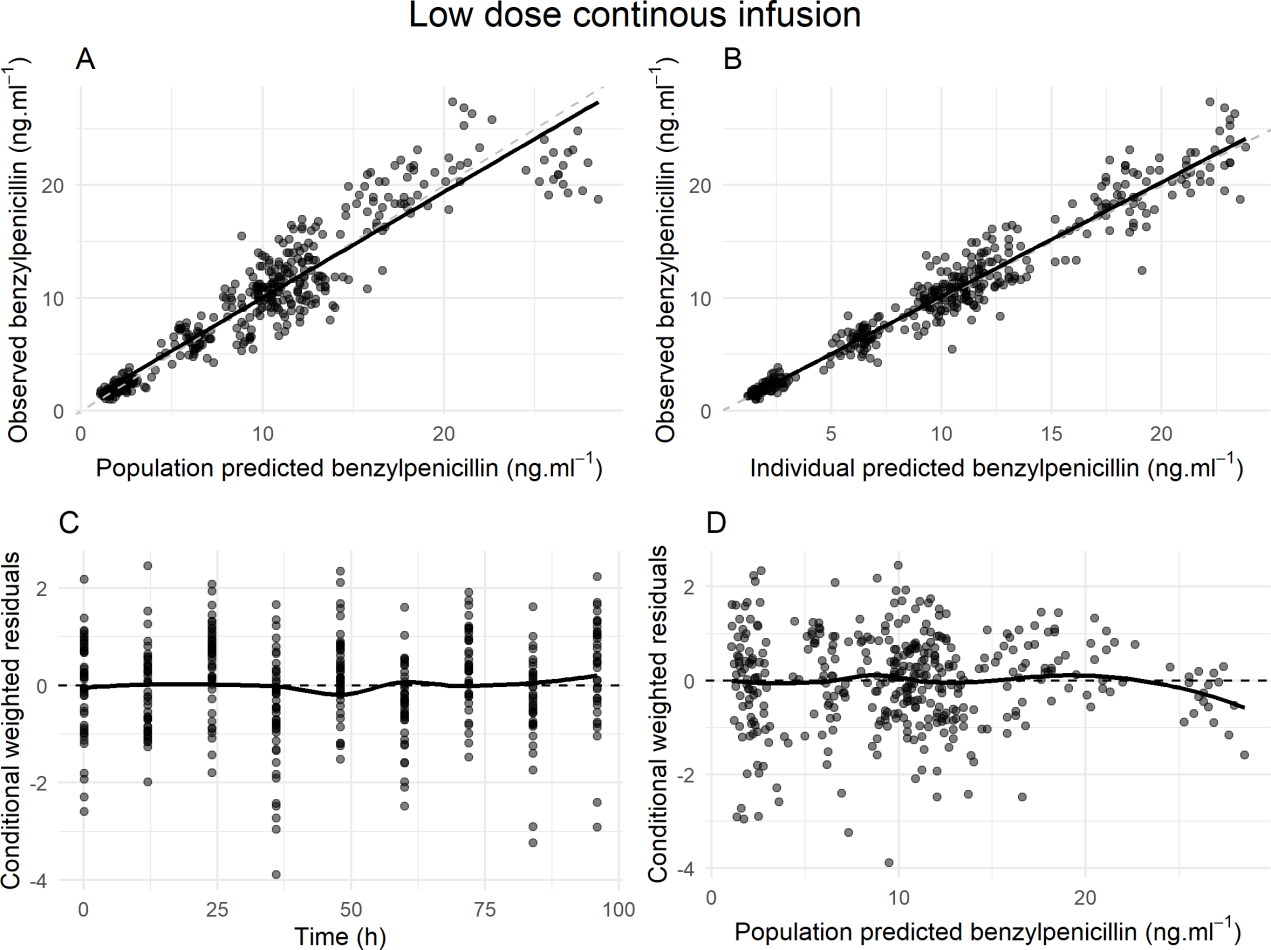
Diagnostic plots for the population pharmacokinetic model of low dose continuous infusion. (**A**) Observed versus population-predicted benzylpenicillin concentrations, (**B**) Observed versus individual-predicted benzylpenicillin concentrations, (**C**) Conditional weighted residuals versus time, and (**D**) Conditional weighted residuals versus population-predicted concentrations. The dashed lines represent the lines of identity, whereas the solid lines indicate the lines of best fit. The low-dose continuous infusion rate ranged from 46.9 to 1133.6  µg/h.

Figure 5’s GOF plots indicate that the model performs well in predicting benzylpenicillin concentrations for the low-dose continuous infusion group. Observed versus predicted plots (Fig. 5A and B) show strong agreement, with minimal deviations. Residual analysis (Fig. 5Cand D) reveals no major biases over time, with residuals centered around zero and consistent variability, except for slight negative residuals at higher concentrations. However, the VPC plot showed good predictions across all target Css values. Overall, the model demonstrates good predictive performance, effectively capturing both population and individual levels.

## DISCUSSION

The CHIPS trial was a randomized, placebo-controlled human challenge study investigating the minimum plasma penicillin concentration needed to prevent Streptococcus pyogenes pharyngitis. In this trial, healthy adults received continuous penicillin infusions targeting specific steady-state concentrations before being experimentally infected with emm75 S. pyogenes. The infusion dose was determined by a preceding single-dose study, in which each participant received a 600  mg bolus infusion over 3  min to establish individual clearance parameters; this clearance value was then used to calculate the continuous infusion dose needed to achieve the targeted concentration. The primary objective of CHIPS was to define a protective Css against S. pyogenes pharyngitis. Ultimately, the goal is to inform future development of long-acting penicillin formulations for preventing recurrent rheumatic fever and rheumatic heart disease. Safety and efficacy data for CHIPS have been published in the main paper (5). However, at the very low target Css of approximately 3 ng/mL (5), the observed clearance was higher than the predictions derived from the preceding single-dose study. EDA suggested that this discrepancy may be related to the low infusion rate—less than 100 µg/h—which arises from multiplying the individual clearance by the target Css. Although EDA indicated a trend toward higher clearance at an infusion rate below 100 µg/h, the population pharmacokinetic analysis did not fully support this finding, possibly due to the wide range of administered doses. The high-dose infusion rate reached about 11,259,260 µg/h, whereas the low-dose infusion rates ranged from 46.88 to 1,133.64 µg/h, producing nearly a 1,000-fold variability in plasma concentrations within a single data set. Consequently, any difference in clearance at such a low infusion rate was difficult to precisely characterize. Instead, when a categorical variable was used for the target Css, the model identified a step increase in clearance at a target Css of 3 ng/mL. The model also suggested that benzylpenicillin exhibits a relatively large volume of distribution at low-dose continuous infusion. Our model’s predicted total clearance of 23 L/h for BSA of 1.73 m^2^ and eGFRcys of 60 mL/min closely aligns with a previous study reporting an average total clearance of 487.4 ± 100.5 mL/min (29 ± 6 L/h) in seven healthy volunteers (four males and three females) aged 21–23 years (7). Our model did not optimally capture the distribution phase of benzylpenicillin, particularly prior to 15 min because our first sampling time was at the 15 min mark. Consequently, the VPC and GOF plots showed a degree of model misspecification in the distribution phase and suggest that benzylpenicillin may distribute more slowly than predicted. However, this finding has minimal clinical significance.

There was a higher volume of distribution in the low-dose continuous infusion. Differences in fluid administration between high- and low-dose infusions may partly help explain the observed discrepancy. Studies in piglets indicate that administering a constant intravenous fluid rate of 6 mL/kg/h can increase the clearance of renally excreted drugs, with the volume of distribution varying according to the specific drug administered (15). In our study, the low-dose groups received a larger total infusion volume (20 mL/h) compared with the high-dose group (a single 10 mL bolus). Although our infusion rate is much lower than in the piglet study, this discrepancy may still partly help explain why the estimated Vc was 1.96 times greater in the low-dose group. However, this difference is not fully explained by fluid administration alone, suggesting that other factors may also be involved. The stepwise increase in clearance is observed only at target Css below 3 ng/mL, and its explanation is largely theoretical. At target Css below 3 ng/mL, the system might remain well below saturation of low-affinity binding sites of albumin, potentially reducing the extent of plasma protein binding and contributing to the observed increase in clearance.

Protein binding of benzylpenicillin is reported to be around 53% (SD 7.7%) (3), particularly to albumin, due to its lysine richness (16, 17). Albumin has both high-affinity and lower-affinity binding sites that can accommodate a range of molecules (18). Benzylpenicillin may occupy several of these sites, including low-affinity sites. When the plasma concentration is below 3 ng/mL, the low-affinity sites may remain unsaturated, leading to a greater fraction of unbound (free) drugs. This unbound fraction is then cleared through both glomerular filtration and active tubular secretion, which likely explains the step-increased clearance at this concentration. This rise in clearance cannot be explained by the saturation of active tubular secretion at bolus infusion concentrations. Renal clearance of benzylpenicillin is primarily through active tubular secretion, which depends on transporter proteins (19). Saturation of these transporters usually occurs at higher concentrations. Because our study’s maximum concentrations (e.g., 45 mg/L after bolus, 27 µg/L after infusion) did not reach the established saturating threshold (EC_50_ of ~48–93 mg/L) (8, 20), true Michaelis–Menten kinetics were not observed. Therefore, the enhanced clearance at 3 ng/mL plasma concentration is more likely attributable to the higher unbound fraction and potentially reduced tubular reabsorption, rather than active transporter saturation.

Tubular reabsorption constitutes a third renal pathway that can modulate the overall clearance of benzylpenicillin. Although benzylpenicillin is largely excreted through active tubular secretion, some reabsorption may occur in the collecting ducts, especially under conditions of low urine flow (21). In a healthy 70 kg, 20-year-old adult, approximately 20%–25% of the cardiac output (around 1.1 L/min) reaches the kidneys, and about 10% of this volume is filtered at the glomerulus (22). This equates to a GFR of roughly 120 mL/min (7.2 L/h) (22). However, because most of the filtered water is reabsorbed, the final urine flow is only 1–2 mL/min (22). This extensive reabsorption of water along the renal tubule serves as the primary driving force for the tubular reabsorption of drugs. Reabsorption is generally concentration-dependent: higher concentrations of benzylpenicillin in the tubular fluid favor reabsorption, whereas lower concentrations lead to less reabsorption. Additionally, passive reabsorption depends on factors such as the drug’s ionization state, urine flow, and urine pH (23). At higher benzylpenicillin concentrations, a stronger concentration gradient enhances reabsorption, and the urine pH tends to be lower due to the drug’s acidic nature, causing more of the drug to remain unionized and thus more readily reabsorbed. In contrast, at very low concentrations, the urine pH is comparatively higher, leaving the drug predominantly in an ionized form that is less likely to be reabsorbed, and the reduced concentration gradient further diminishes reabsorption.

Collectively, these mechanisms—particularly an increased unbound fraction—offer a plausible explanation for the unexpected step increase in clearance of benzylpenicillin at Css below 3 ng/mL.

The measured renal clearance of benzylpenicillin (309.4 mL/min, ~18.5 L/h) in healthy volunteers (7) falls between the GFR and renal plasma flow, underscoring the importance of both filtration and active secretion in its elimination. Notably, tubular secretion does not always decline proportionally to GFR (24), meaning that GFR-based estimates alone may misrepresent true drug clearance in certain populations. Nevertheless, our study population consisted of healthy volunteers with normal renal function. We found that using eGFR estimates for clearance based on cystatin C correlated better with actual benzylpenicillin clearance than did creatinine-based equations. This finding aligns with previous studies where cystatin C was found to be a superior predictor of antibiotic clearance (e.g., vancomycin, ceftriaxone, and cefepime) compared with creatinine (14, 25, 26) largely because cystatin C levels are less influenced by factors such as age, sex, and muscle mass (27). Our results suggest that cystatin C-based eGFR may also be more accurate for guiding benzylpenicillin dosing.

This study is not without limitations. It was initially designed following the CHIPS clinical protocol, and an unexpected increase in clearance below 3  ng/mL was observed. Although the analysis quantitatively identified a step increase in clearance at this threshold, it may be more plausible that the change occurs more gradually around 3  ng/mL. Further investigation at these very low doses could provide more precise quantification, but the clinical significance of such granular detail remains uncertain.

In summary, the unexpectedly high clearance of benzylpenicillin at Css of 3 ng/mL can potentially be explained by a combination of factors, including diminished plasma protein binding saturation and decreased tubular reabsorption. In addition, cystatin C-based eGFR appears to more accurately predict benzylpenicillin clearance, potentially enabling more precise individualized dosing in clinical practice.

This study observed that as plasma concentrations approached approximately 3  ng/mL, penicillin levels rapidly declined. At the next assessed concentration of 6  ng/mL, a more gradual reduction in plasma levels was observed. These findings suggest that maintaining penicillin concentrations above 6  ng/mL may facilitate more controlled clearance. To avoid abrupt declines and achieve more stable pharmacokinetic behavior, the dosing interval should be guided such that the next dose is administered just before the plasma concentration reaches the trough level of 6 ng/mL. A new long-acting formulation should be designed to maintain zero-order release, ensuring plasma concentrations remain above 6 ng/mL to prevent a sudden drop in drug levels.

## MATERIALS AND METHODS

### Participants

This population pharmacokinetic study was conducted as part of a previously published double-blinded, placebo-controlled randomized clinical trial to determine the minimum concentration of benzylpenicillin required to prevent experimental pharyngitis with *Streptococcus pyogenes* (the CHIPS Trial) (28). Healthy adult males and non-pregnant, non-lactating females aged 18–40 years without pre-existing risk factors for severe *S. pyogenes* disease were recruited (28). The study received approval from the Bellberry Human Research Ethics Committee, Australia (2021–03-295), and written informed consent was obtained from all participants. A safety review committee—comprising an independent chair, an infectious disease expert, and a biostatistician—evaluated safety data and performed pre-planned interim analyses between each cohort of 15 participants. The trial took place in Perth, Australia, between August 2022 and July 2023. Participants received remuneration at a level approved by the ethics committee, commensurate with the loss of income associated with trial participation.

### Study design

Between 7 and 35 days prior to randomization, each participant underwent a single-dose PK evaluation of intravenous benzylpenicillin. Each participant received a single 600 mg dose administered intravenously over 3 min in a volume of 10 mL (28). Blood samples were collected at 0.25, 0.5, 1, 2, 3, 4, and 6 h post-dose. These samples were collected in EDTA tubes, and centrifuged, and the plasma was stored at –80  °C. Penicillin concentrations in plasma were measured using a validated liquid chromatography-mass spectrometry assay within 24 h (29). Individual clearance and volume of distribution estimates were generated using NONMEM (version 7.2.0, ICON Development Solutions, Ellicott City, MD, USA) employing the First-Order (FO) method, with benzylpenicillin plasma concentration-time data.

Participants were then randomized to receive low-dose continuous infusions aimed at achieving target Css of 0, 3, 6, 9, 12, or 20 ng/mL. The loading and continuous infusion doses required to achieve these allocated Css values were calculated for each participant, based on individual estimates of the volume of distribution and clearance obtained from the single 600 mg IV bolus PK assessment. After admission, participants were confined to a clinical trials unit and received the allocated infusion through a midline intravenous catheter. Steady-state plasma benzylpenicillin concentrations were measured every 12 h after the infusion started. The infusion bags were changed every 12 h, and 5 mL of the infusion solution was sampled and analyzed to determine the actual benzylpenicillin concentration. Blood samples were processed and stored in the same manner as those from the bolus dose. Infusion bag samples were also stored at –80  °C. All samples were analyzed together after the completion of sample collection, following approximately 1 year of storage. All participants were monitored closely for adverse events for up to 5 days. The primary endpoint was the development of clinical pharyngitis following controlled experimental infection with *S. pyogenes*.

Infusion bags were prepared by an independent compounding pharmacist at randomization and transported to the inpatient facility with blinded labeling (28). The stability of the citrate-buffered benzylpenicillin preparations under simulated trial conditions has previously been demonstrated (30). Penicillin concentrations in plasma and infusion bags were measured using a validated liquid chromatography-mass spectrometry assay (29). Actual infusion rates, as confirmed by these measurements, were used in the pharmacokinetic analyses.

### Exploratory data analysis

The clearance values of low-dose infusion were calculated according to equation 1:


(1) Infusion rate=Clearance×Css

The relationship between clearances from the bolus and low-dose infusion data was plotted using R version 4.1.3 (R Foundation for Statistical Computing, Vienna, Austria).

### Population pharmacokinetic analysis

Combined data from both the bolus and continuous infusion phases were subsequently subjected to population PK analysis using NONMEM (v 7.5.1, ICON Development Solutions, Ellicott City, MD, USA) with a GFortran 4.6.0 compiler, supported by Perl-Speaks-NONMEM (PsN) and Pirana (31). The first-order conditional estimates method with interaction (FOCE INTER) was employed to fit the log-transformed plasma concentration–time data, guided by the minimum OFV, GOFs, and VPCs. Nested models were compared using a significance level of *P* < 0.05.

The base structural models included the volume of distribution of the central compartment (Vc), clearance (CL), peripheral volume(s) of distribution (Vp), and intercompartmental clearance(s) (Q). One-, two-, and three-compartment models (ADVAN1, −3, and −11) with first-order elimination from the central compartment were compared. Once the model structure was finalized, interindividual variability (IIV) of parameters was estimated where supported by the data. Allometric scaling based on body size was applied a priori, with exponents of 0.75 for Q and 1 for volume parameters. Zero-order elimination combined with first-order kinetics was also evaluated.

Ratio terms distinguishing bolus and low-dose continuous infusion were tested on clearance, V1, or V2. An additive difference in clearance or V1 estimates between the bolus and low-dose continuous infusion was also examined, as were covariates such as body weight, BSA, age, and sex. Various error models were tested, including additive, combined additive/proportional, and separate error models for low concentrations. Subsequently, the effect of renal function on clearance was evaluated.

### Benzylpenicillin clearance predictor

Cystatin C or serum creatinine was measured within 1 week prior to the infusion, using enzymatic and turbidometry methodology, respectively, accredited by the National Association of Testing Authorities (NATA). Renal function was estimated using creatinine clearance (Cockcroft–Gault) with actual body weight (CLCr) (32) and the estimated glomerular filtration rate (eGFR) from:

1. The CKD-EPI Cystatin C Age, Sex Equation (2012) (33) (eGFRcys)
2. The CKD-EPI Creatinine Age, Sex Equation (2021) (34). (eGFRcr)
3. The CKD-EPI Creatinine-Cystatin C Age, Sex Equation (2021) (34). (eGFRcr-cys) eGFR values (mL/min/1.73 m^2^) were converted to mL/min using the participant’s body surface area (BSA), calculated using equation 2:

(2) $$\text{BSA}=\sqrt{Height\;\left(cm\right)\times Weight\left(kg\right)/3600}$$

Clearance was modeled as a function of individual renal function using eGFR (expressed in mL/min, then divided by 60 mL/min). The resulting OFVs were compared to determine the most appropriate model specification.

### Model evaluation

GOFs included observed versus individual and population predictions, as well as residual plots against time from the first dose and against population predictions. A bootstrap analysis with 1,000 replicates was conducted using PSN, and parameter estimates were summarized as medians with 2.5th and 97.5th percentiles (95% empirical confidence interval). In addition, prediction-corrected visual predictive checks (pcVPC) were generated from 1,000 simulated data sets based on the final model to further evaluate its predictive performance.
